# Tamoxifen: the drug that came in from the cold

**DOI:** 10.1038/sj.bjc.6605231

**Published:** 2009-08-11

**Authors:** L Hughes-Davies, C Caldas, G C Wishart

**Affiliations:** 1 Cambridge Breast Unit, Addenbrooke's Hospital, Hills Road, Cambridge, UK; 2 Department of Oncology, University of Cambridge, and Functional Breast Cancer Genomics Laboratory, Cancer Research UK Cambridge Research Institute, Li Ka Shing Centre, Robinson Way, Cambridge CB2 0RE, UK; 3 NIHR Cambridge Biomedical Research Centre, Cambridge, UK

**Keywords:** breast cancer, adjuvant therapy, tamoxifen, aromatase inhibitors, oestrogen receptor, post-menopausal

## Abstract

Despite the perception of many oncologists that tamoxifen is an inferior drug, and should be substituted by an aromatase inhibitor in post-menopausal women, the current evidence strongly supports the view that AIs should be used 2–3 years after tamoxifen to achieve the maximal overall survival (OS) advantage.

The last year has been an interesting time for oncologists interested in the adjuvant hormonal treatment of post-menopausal women with receptor-positive early breast cancer. Three important new pieces of clinical research were presented at the 2008 San Antonio Breast Cancer Symposium: a meta-analysis of the Aromatase Inhibitor (AI) trials (Ingle *et al*, 2008b) and two individual AI studies (Jakesz *et al*, 2008; Mouridsen *et al*, 2008). These data suggest that it may be premature for oncologists to discard tamoxifen. In this mini review, we analyse whether all patients should be exposed to 2 or 3 years of tamoxifen as part of their adjuvant hormone therapy for receptor-positive early-stage breast cancer.

Improved mortality from breast cancer during the past two decades has been partly attributed to increased use of adjuvant hormonal treatment. The Oxford Overviews showed that tamoxifen, an oestrogen receptor ligand, can halve the risk of recurrence and reduce the risk of death by about a quarter in oestrogen receptor positive patients (EBCTCG, 2005). Benefit is seen in all patient groups, regardless of age, stage or whether chemotherapy has been used. The evidence for a mortality benefit from tamoxifen led to a search for better adjuvant hormonal treatments. The first trials of highly selective AIs in post-menopausal women began to report in 2002 (Baum *et al*, 2002 and Table 1). In contrast to tamoxifen, which binds to the oestrogen receptor and modulates its function, the AIs achieve near complete inhibition of oestrone production, the principal oestrogen in post-menopausal women. This deprives oestrogen receptor-positive breast cancer cells of their most important proliferative signal. As the table illustrates, the AI trials have been among the largest yet seen in breast cancer.

The AI trials have three designs: substitution trials, where an aromatase inhibitor is given instead of tamoxifen; switching trials, where 2 to 3 years of tamoxifen and 2 to 3 years of an aromatase inhibitor are given sequentially for a total of 5 years of hormonal treatment (tamoxifen has usually been given first in these trials), and extension trials, where 5 years of tamoxifen treatment is followed by 2 to 3 years of an aromatase inhibitor. Each of these AI trials has clearly shown a disease-free survival (DFS) advantage for the AI. These DFS outcomes were so convincing that by 2005 the ASCO Technology Assessment Panel had ruled that optimal adjuvant hormonal therapy should include an AI, ‘but it remains unclear if initial treatment with an aromatase inhibitor is superior to a planned cross over from tamoxifen’ (Winer *et al*, 2005). Despite the ASCO Panel's careful wording, most oncologists concluded that tamoxifen was an inferior drug and should no longer be used in their post-menopausal patients. Within a few months Patterns of Care surveys showed that over 80% of oncologists in North America had stopped using tamoxifen altogether for post-menopausal patients (Love, 2005). In the light of these new data, it is now time to consider whether this was the correct decision.

There is increasing concern that some of the most influential AI trials have been unable to demonstrate an overall survival (OS) advantage of AI’s compared with tamoxifen particularly when used up front. For example Seruga and Tannock recently reviewed the evidence and concluded that ‘there is no evidence for superiority of AIs over tamoxifen when used as initial treatment’ (Seruga and Tannock, 2009). They were undecided about the possible survival benefit of a switching strategy, conceding only that a recent metanalysis of the switching trials ‘suggests an advantage’ in overall survival. Surprisingly this metanalysis (Jonat *et al*, 2006) did not include the International Exemestanse Study which ‘was excluded because it investigated a steroidal aromatase inhibitor’ (Seruga and Tannock, 2009).

Here, we develop the case put by Seruga and Tannock in the light of fresh evidence, focusing particularly on the case for a switching strategy. Most of the trials included have been updated since the meta-analysis, doubling the median follow up time. We also think that it is legitimate to consider trials that used exemestane, as many oncologists believe that there is little or no clinical difference between steroidal and non-steroidal AIs. We will also consider the statistical pitfalls that confront investigators as they grapple with the problem of crossover. This has affected almost all of the AI trials and has confused their reporting.

The first AI trial to report was the ATAC study, a substitution trial that compares 5 years of anastrozole to 5 years of tamoxifen (ATAC Trialists Group, 2008). The ATAC trial showed a convincing early improvement in disease-free survival for the anastrozole arm, a finding which generated great interest and led to the rapid and widespread changes in practice described above. To date, however, ATAC has failed to show any OS benefit for the AI arm (ATAC Trialists Group, 2008). Initially these concerns were dismissed because it can take time for an OS difference to emerge, but as the follow-up has stretched out to 100 months, this concern has steadily grown. No OS difference has emerged from the ATAC study. Indeed there is not even the suggestion of a trend towards an improved survival advantage.

Two explanations have been offered to explain the lack of an OS benefit in the ATAC trial. First, longer follow up is needed. Second, about two-thirds of the patients enrolled into the ATAC study were node negative, and it has been suggested that their relatively good baseline prognosis makes it harder to demonstrate a significant absolute mortality difference. It is equally possible, however, that the complete substitution of tamoxifen by an AI is not the best way to use these new drugs. Perhaps there are some patients who need tamoxifen for their cure.

For patients in their first consultation after surgery for breast cancer, there is one question, which comes above all others: How can I reduce my risk of death? If we look at all of the AI data with this single question in mind, we can see that mortality benefits are only seen in a subset of trials. We have called these the ‘OS-positive trials’ (highlighted in Table 1). In one trial (the MA 17 trial), the OS advantage was seen in a planned subgroup analysis (Goss *et al*, 2005), but in the other trials this was a prospectively predefined end point seen in the entire study population. It is striking that all of the OS-positive trials included a period of tamoxifen before the AI. Indeed it seems that OS positivity is confined to trials, which used a sequenced tamoxifen-AI strategy, rather than an AI alone.

This conclusion is supported by the meta-analysis of AI trials which was presented at the recent San Antonio meeting (Ingle *et al*, 2008b) which examined all of the AI trial data, and divided patients into two cohorts: those patients who never received tamoxifen and those who received tamoxifen before taking an AI. The first cohort is OS negative, whereas the second is OS positive. Importantly, and in contrast to an earlier metanalysis (Jonat *et al*, 2006), the Ingle metanalysis includes all aromatase inhibitors of either the non-steroidal (letrozole and anastrozole) or steroidal (exemestane) class. It therefore includes the largest switching trial (the International Exemestane Study), which shows a significant overall survival benefit.

One important issue that needs to be addressed in the OS-positive tamoxifen/AI studies is potential bias at the randomisation point. Patients in substitution trials were randomised shortly after diagnosis, whereas those in switching trials were randomised at the switch point. If patients are randomised after they have been on tamoxifen for 2 to 3 years, then a small but important pool of early relapsing patients, with hormonally refractory breast cancer, will have been removed from the patient population. The removal of this poorly responsive group could account for the ability of AIs to achieve a mortality benefit in the more hormonally responsive patients who made it through the first 2 or 3 years to the randomisation point.

The 2008 San Antonio Breast Cancer Symposium included two large studies, which shed some light on this issue. Before considering this more fully, we need to acknowledge that the analysis of some of these trials is complicated by a crossover problem. The early DFS improvement from AIs were widely publicised in the early 2000s. It was therefore inevitable that large numbers of women assigned to tamoxifen in other trials would opt to withdraw from these studies and cross over to an AI. Investigators have attempted to deal with the crossover problem by analysing their data in three different ways, Intent to Treat (ITT), censored or retrospective. ITT analysis ignores the cross over and keeps patients in their originally assigned treatment groups, regardless of whether they are actually taking tamoxifen or not. This is a statistically rigorous approach, but it may contribute to an underestimation of the benefits of AIs. The ITT method therefore may be less informative and less persuasive for changing clinical practice. Alternatively, censored data can be used. Using the Kaplan–Meier method, patients are silently removed from the data set (censored) at the moment that they withdraw themselves from the control arm of the trial to take an AI. The third approach is the retrospective analysis in which patients are analysed by what drug they received rather than how they were initially assigned.

Although censored or retrospective analysis seems to deal with the problem of cross over, these approaches become problematic if there is any association between a woman's desire to leave the control arm of a trial and her risk of relapse or death. In fact, such an association is quite likely. For example, one can easily imagine that a younger woman at higher risk might take more interest in breast cancer research, including the emerging results from the ATAC trial, and have a lower threshold for asking to come out of trial and cross over to the AI arm, whereas older women with lower risk disease and other comorbidites might be content to remain on the original assigned treatment.

There is now strong evidence that exactly this kind of crossover bias does exist. When the MA17 trial reported a DFS advantage for letrozole (Goss *et al*, 2005), patients in the control arm were informed and offered the option of crossing over. About two-thirds of the placebo patients opted to cross over. The clinical course of these placebo patients was recently reported in an update paper which has become known as the MA17 Unblinding Study (Goss *et al*, 2008). This fascinating and detailed study of a mass crossover event in a large contemporary randomised study offers deep insights into the many sources of bias which could contaminate a censored or retrospective analysis. Patients who opted for the cross over were substantially younger, more likely to have received chemotherapy and were more frequently node positive (Goss *et al*, 2008). These multiple biases are likely to act in different directions and therefore make a censored analysis problematic. On one hand, higher risk patients tended to cross over to the investigational treatment (which favours the control arm). On the other, older patients tend to prefer to stay on their assigned control treatment, which would strongly affect the OS curves (in favour of the investigational treatment).

That these sources of bias are powerful, and can lead to results which are clearly artefactual, is also shown in the MA17 Unblinding Study (Goss *et al*, 2008). The retrospective analysis of outcome generated a highly significant hazard ratio for OS of 0.3 (0.17–0.53) in favour of letrozole. This mortality result is better than anything we have seen in any therapeutic breast cancer trial over the past half century, and must simply reflect the older age of the patients who decided not to cross over rather than any unprecedented lifesaving effect of letrozole. For these reasons, we believe that oncologists should not be seduced into using the (apparently reasonable) censored or retrospective analyses of trials, which are so often seen as an investigator's attempt to salvage trials with a high crossover rate. As a result, ITT (Intention to Treat) is the only appropriate method that should be used to analyse the current AI trials.

The larger of the two trials to report at San Antonio 2008 was the BIG 1–98 study (Mouridsen *et al*, 2008), a complex trial which compares both a switching and substitution design to the standard 5 years of tamoxifen. In the ITT analysis, no statistically significant OS difference was seen between the two monotherapy arms (tamoxifen alone *vs* letrozole alone) at 76 months. This trial also included two switching arms, but OS data has not yet been reported for this part of the trial, so this is not included in the table. These latest results from BIG 1–98 reinforce the pattern previously described: OS positivity is not seen when AIs are given up front. We predict that an overall survival difference will eventually be seen in this trial and this will favour a switching strategy.

The Austrian Breast Cancer Study Group (ABCSG8) trial was also reported at San Antonio and is one of the most interesting of the adjuvant AI trials because it shows a clear OS benefit with shorter follow up than ATAC in a patient population that is similar. The crossover rate from the tamoxifen arm in this trial was less than 6%, so the use of a censored method may be legitimate and, the survival advantage remains significant even if data are subjected to an ITT analysis (R Jakesz, personal communcation). ABCSG8 undermines the arguments that have been used to explain away the lack of a mortality benefit in ATAC. OS positivity was clearly seen at 72 months, which is 2 years earlier than the ATAC follow up. Furthermore, the node positivity rates of the patients in the ABCSG trial are similar to those of ATAC. The mortality benefits from ABCSG8 suggest that if we treat 60 patients for 6 years, one life will be saved. It is now the fourth OS-positive AI trial. All four of the OS-positive AI trials have been switching trials. Or in other words, mortality benefits are only seen in trials, which give AIs after an initial period of tamoxifen.

In our view, oncologists should always remember the question that comes before all others. How can an individual patient reduce her risk of death? The available evidence for receptor-positive post-menopausal women strongly supports the use of an approach in which all patients are exposed to 2–3 years of tamoxifen and 2–3 years of an aromatase inhibitor. All OS-positive trials use such an approach and this is the only treatment strategy for which a mortality benefit could be seen in the AI meta-analysis. Such an approach also limits the patient's exposure to either class of drug. As these two classes of drug have different safety profiles (tamoxifen is associated with small risks of endometrial cancer and thromboembolic disease whereas the AI's have a moderate effect on bone health) this additional limited exposure to either agent is likely to mitigate the risk of serious complications.

## Conclusion

Recent trial data support the view that AIs should be used after 2–3 years of tamoxifen to achieve an OS advantage and add weight to scepticism surrounding up front use of these drugs (Seruga and Tannock, 2009), a strategy which we believe should only be used for women with contraindications to tamoxifen.

## Figures and Tables

**Table 1:** A summary of the AI trials that have been reported to date

	***n***	**AI**	**Design**	**Age**	**ER+**	**% *n*+**	**FU**	**DFS HR**	**Events AI/TAM**	**DFS P val**	**OS**	**Deaths AI/TAM**	**OS P val**	**ref**
ATAC	5216	A	sub	64	100	35	100	0.85	618/702	0.003	0.97	472/477	NS	1
BIG 1-98	4922	L	sub	61	98	41	76	0.88	509/565	0.03	0.87	303/343	0.08	2
IES	4602	E	sw	n/s	100	48	56	0.75	339/439	0.0001	0.83	210/251	0.05	3
ABCSG-8	2922	A	sw	n/s	100	25	72	0.79	202/235	0.038	0.77	130/157	0.025	4
ARNO-95	979	A	sw	61	97	25	30	0.66	36/47	0.049	0.53	15/28	0.045	5
ITA/GROCTA	828	A,AG	sw	64	100	85	78		not stated		0.61	48/74	0.007	6
NSABP B33	1598	E	ext	60	100	48	30	0.68	37/52	0.07	1.0	16/13	NS	7
MA17	5187	L	ext	62	97	46	64	0.68	164/235	0.0001	1.0	154/155	NS	8

Abbreviations: sub=substitution, sw=switching, ext=extension. A=Anastrozole, L=Letrozole, E=Exemestane, AG=Aminogluthemide.

Those trials that have a statistically significant mortality benefit are highlighted in pink. This table clearly shows that only the switching trials have been able to show a mortality benefit from the AIs.

Refs: 1=ATAC Trialists, 2008; 2=Mouridsen *et al*, 2008; 3=Coombes *et al*, 2007; 4=Jakesz *et al*, 2008; 5=Kaufmann *et al*, 2007; 6=Boccardo *et al*, 2007; 7=Mamounas *et al*, 2008; 8=Ingle *et al*, 2008a.

All these analyses are intent to treat (ITT) with no adjustment for crossover and patients are kept in their originally assigned groups even if they crossed over from the control arm to the investigational arm. However, for the ATAC and IES data presented in this table, the ER unknown or ER-negative patients are excluded from analysis.

For MA17, a 2005 report showed an OS advantage in the node-positive subgroup; this was not seen in the most recent results, probably because of a high crossover rate.

For ATAC, the figure in the table is for the ER-positive subgroup.
